# Adverse Drug Reactions: Building Drug Safety Beyond Marketing Authorisation

**DOI:** 10.3390/jcm15186994

**Published:** 2026-09-10

**Authors:** Andrej Belančić, Ivana Mudnić

**Affiliations:** 1 Department of Basic and Clinical Pharmacology and Toxicology, Faculty of Medicine, University of Rijeka, Braće Branchetta 20, 51000 Rijeka, Croatia; 2 Department of Basic and Clinical Pharmacology, University of Split School of Medicine, Šoltanska 2A, 21000 Split, Croatia; ivana.mudnic@mefst.hr or ivancicamudnic@gmail.com

Marketing authorisation is a regulatory conclusion that, on the basis of the available evidence, the medicine has a favourable benefit–risk balance for a defined population and indication. No pre-authorisation development programme can reproduce the full diversity of patients, comorbidities, concomitant treatments, durations of exposure, and patterns of use encountered in clinical practice, nor can it reliably identify all rare or delayed adverse reactions [1]. Thus, the question is not whether uncertainty remains when a medicine enters clinical practice, but how efficiently and rigorously that uncertainty can subsequently be reduced.

Adverse drug reactions remain an important consequence of pharmacotherapy and a continuing challenge for clinical practice and clinical pharmacology. They may compromise treatment effectiveness, result in additional health-care utilisation, prolong hospitalisation, impair quality of life, or, in severe cases, cause permanent disability or death. Their recognition can be straightforward when the temporal relationship and clinical phenotype are characteristic, but considerably more difficult when a reaction is rare, delayed, immune-mediated, idiosyncratic, or clinically indistinguishable from the underlying disease [2]. Drug safety is therefore not simply a matter of cataloguing adverse effects. It is a continuous process of detecting, characterising, interpreting, and acting on evolving evidence and uncertainty [3].

This Special Issue of the Journal of Clinical Medicine brings together contemporary research addressing these different dimensions of drug safety. The contributions span pharmacovigilance and disproportionality analyses of large spontaneous-reporting databases, real-world evidence from electronic health records, retrospective clinical investigations, case series, and narrative reviews. They address drug-induced renal injury [4,5], haematological [6] and immune-mediated adverse events [7], hypersensitivity reactions [8,9], electrolyte disturbances [10], dermatological toxicity, and serious rare reactions such as Stevens–Johnson syndrome [11]. They also examine safety considerations associated with contemporary therapies, including immune checkpoint inhibitors [7], metabolic therapies [12,13], antimicrobials [4,8], proton pump inhibitors [5], isotretinoin [14], and treatments for rare diseases such as spinal muscular atrophy [15]. Although diverse in subject matter, these contributions converge on a common principle: the safety of a medicine is progressively learned through its use, and this learning must continue long after regulatory authorisation [16].

Spontaneous adverse-event reporting remains one of the foundations of this process. Its principal strength is breadth: reports arising from routine clinical practice can reveal unexpected patterns, including rare events that may be difficult to identify reliably during pre-authorisation development. Disproportionality analysis can provide an important early indication that an adverse event is reported disproportionately with a particular medicine compared with a defined reporting background. However, a statistical signal is not a diagnosis of causality. Reporting behaviour, concomitant medicines, underlying disease, differential exposure, stimulated reporting, and other sources of bias can influence observed associations [17]. The appropriate response to a signal is therefore not automatic attribution, but structured assessment and, where necessary, further investigation.

The contributions to this Special Issue demonstrate how such signals can be examined across different organ systems and therapeutic classes. Analyses of spontaneous-reporting data address renal, haematological, immune, electrolyte, and dermatological adverse events, while other work focuses on serious and potentially life-threatening reactions. Together, these manuscripts illustrate both the continuing value of pharmacovigilance databases and the need to interpret their findings within a broader evidentiary framework [16].

That broader framework is increasingly supported by real-world evidence. Once a medicine is used at scale, electronic health records, claims data, registries, and other routinely collected health data can provide information on populations, exposures, comorbidities, concomitant treatments, laboratory parameters, hospitalisations, and outcomes that are difficult to capture comprehensively in clinical trials. Such data can help determine whether a signal observed in spontaneous reports is reproducible, quantify risks in defined populations, and identify circumstances in which an adverse reaction is more likely to occur. Yet larger datasets do not automatically produce more reliable evidence. Confounding, misclassification, missing data, differential surveillance, selection effects, and other sources of bias remain important. The value of real-world evidence depends on the fitness of the data for the research question, the methodological design, and the clinical interpretation as much as on data volume [18,19].

The need for complementary evidence is particularly apparent in populations and clinical situations that are incompletely represented in trials. Older adults, patients with multiple comorbidities or polypharmacy, and individuals with organ dysfunction may have risk profiles that differ from those observed in selected trial populations. Similarly, the broader and longer exposure that follows widespread clinical use can reveal safety issues that were not apparent during development. For rare diseases, where the underlying populations are small by definition, the challenge is greater still. Drug safety must, therefore, be understood as a process of continuous evidence generation rather than as a finite assessment completed at the point of authorisation. This principle is already embedded in regulatory pharmacovigilance, which requires ongoing evaluation of benefit–risk balance and, when necessary, additional post-authorisation studies and risk-management measures [15,18,19].

The same principle applies to drug hypersensitivity and related clinical decision-making. Recognising a suspected reaction is only the first step. Determining whether the medicine was responsible, assessing the likelihood of cross-reactivity, deciding whether an alternative treatment is genuinely necessary, and identifying patients who may safely undergo appropriate allergy evaluation or supervised drug challenge can have direct consequences for future treatment. An inaccurate drug-allergy label can unnecessarily narrow therapeutic options, whereas failure to recognise a genuine severe reaction can expose a patient to substantial risk. Biomarkers, diagnostic tests, structured clinical assessments, and carefully selected re-exposure strategies may therefore transform drug safety from retrospective description into prospective risk management [20].

This shift from detecting harm to preventing harm is also a major opportunity offered by modern health-data infrastructure. The period immediately after marketing authorisation should not be regarded as a phase during which pharmacovigilance must rely primarily on the accumulation of spontaneous reports. Contemporary pharmacovigilance already incorporates signal detection and assessment, periodic evaluation of benefit–risk balance, risk-management activities, and post-authorisation studies. The opportunity now is to complement these established approaches with systematic analyses of routinely collected health data from the earliest meaningful exposures. Health-care systems already generate longitudinal information on prescriptions, diagnoses, laboratory results, hospitalisations, and outcomes. When these data can be analysed using prespecified outcomes, appropriate comparators, and robust methodological safeguards, surveillance can become more timely and more informative.

Rapid analysis without methodological discipline risks generating false-positive associations, diverting resources, and undermining confidence in pharmacovigilance. Prespecified protocols, appropriate comparators, negative controls where applicable, sensitivity analyses, careful assessment of confounding and bias, and evaluation of clinical plausibility are essential if faster evidence generation is to remain trustworthy. Recent methodological developments and regulatory guidance increasingly support the use of real-world data for non-interventional safety studies, but they also emphasise the need for explicit research questions, fit-for-purpose data sources, robust variable definitions, and appropriate approaches to bias and confounding [18,19,21].

Artificial intelligence and other computational approaches may further accelerate the extraction, linkage, classification, and synthesis of safety information from structured and unstructured clinical data. Their role, however, should be to augment, not to replace clinical pharmacology expertise and human assessment. The objective is not merely to identify more associations, but to distinguish clinically meaningful signals from artefacts, understand mechanisms and risk factors, and translate evidence into decisions that improve patient care [22].

The contributions assembled in this Special Issue consequently represent different components of the same endeavour. Spontaneous reports can identify potential signals; real-world studies can test and quantify them; clinical investigations can characterise phenotypes and risk factors; diagnostic and biomarker approaches can improve recognition; and clinical pharmacology can translate the resulting evidence into decisions about monitoring, treatment continuation, modification, avoidance, or, where appropriate, re-exposure. Regulatory pharmacovigilance provides the framework within which these sources are integrated and acted upon.

The future of drug safety should therefore not be defined by the aspiration to eliminate uncertainty at the moment of marketing authorisation, a goal that is neither realistic nor scientifically necessary, but by the ability to reduce uncertainty rapidly, systematically, and responsibly once a medicine enters clinical practice. Many of the technological and methodological foundations for more proactive surveillance now exist. The remaining challenge is to integrate them effectively into clinical, regulatory, and health-care systems—to treat the post-authorisation safety profile of every medicine as knowledge that must be actively built, tested, updated, and communicated, rather than knowledge that can simply be awaited.

For clinical pharmacology, this is both an opportunity and a responsibility. The ultimate purpose of pharmacovigilance is not to generate the largest number of signals, publications, or statistical associations. It is to recognise clinically meaningful harm earlier, identify patients at greatest risk, prevent avoidable exposure, and preserve effective treatment whenever possible [23]. The central question is therefore not merely “Can this medicine cause harm?”, but “What have we learned about that harm, how certain are we, and what should change for the patient in front of us?”

This Special Issue contributes to that continuing process of learning—moving drug safety from signal detection toward active understanding, and from recognition of adverse reactions toward their prevention and better management.
